# Functional evaluation of rare variants in complement factor I using a minigene assay

**DOI:** 10.3389/fimmu.2024.1446081

**Published:** 2024-08-22

**Authors:** Cobey J. H. Donelson, Nicolo Ghiringhelli Borsa, Amanda O. Taylor, Richard J. H. Smith, Yuzhou Zhang

**Affiliations:** Molecular Otolaryngology and Renal Research Laboratory, Carver College of Medicine, University of Iowa, Iowa City, IA, United States

**Keywords:** complement, alternative pathway, complement factor I, complement-mediated diseases, haploinsufficiency, RNA splicing, Cis/trans-acting elements

## Abstract

The regulatory serine protease, complement factor I (FI), in conjunction with one of its cofactors (FH, C4BP, MCP, or CR1), plays an essential role in controlling complement activity through inactivation of C3b and C4b. The functional impact by missense variants in the *CFI* gene, particularly those with minor allele frequencies of 0.01% to 0.1%, is infrequently studied. As such, these variants are typically classified as variants of uncertain significance (VUS) when they are identified by clinical testing. Herein, we utilized a minigene splicing assay to assess the functional impact of 36 ultra-rare variants of *CFI*. These variants were selected based on their minor allele frequencies (MAF) and their association with low-normal FI levels. Four variants lead to aberrant splicing–one 5’ consensus splice site (NM_000204.5: c.1429G>C, p.Asp477His) and three exonic changes (c.355G>A, p.Gly119Arg; c.472G>A, p.Gly158Arg; and c.950G>A, p.Arg317Gln)–enabling their reclassification to likely pathogenic (LP) or pathogenic (P) based on ACMG guidelines. These findings underscore the value of functional assays, such as the minigene assay, in assessing the clinical relevance of rare variants in *CFI*.

## Introduction

**Figure 1:**
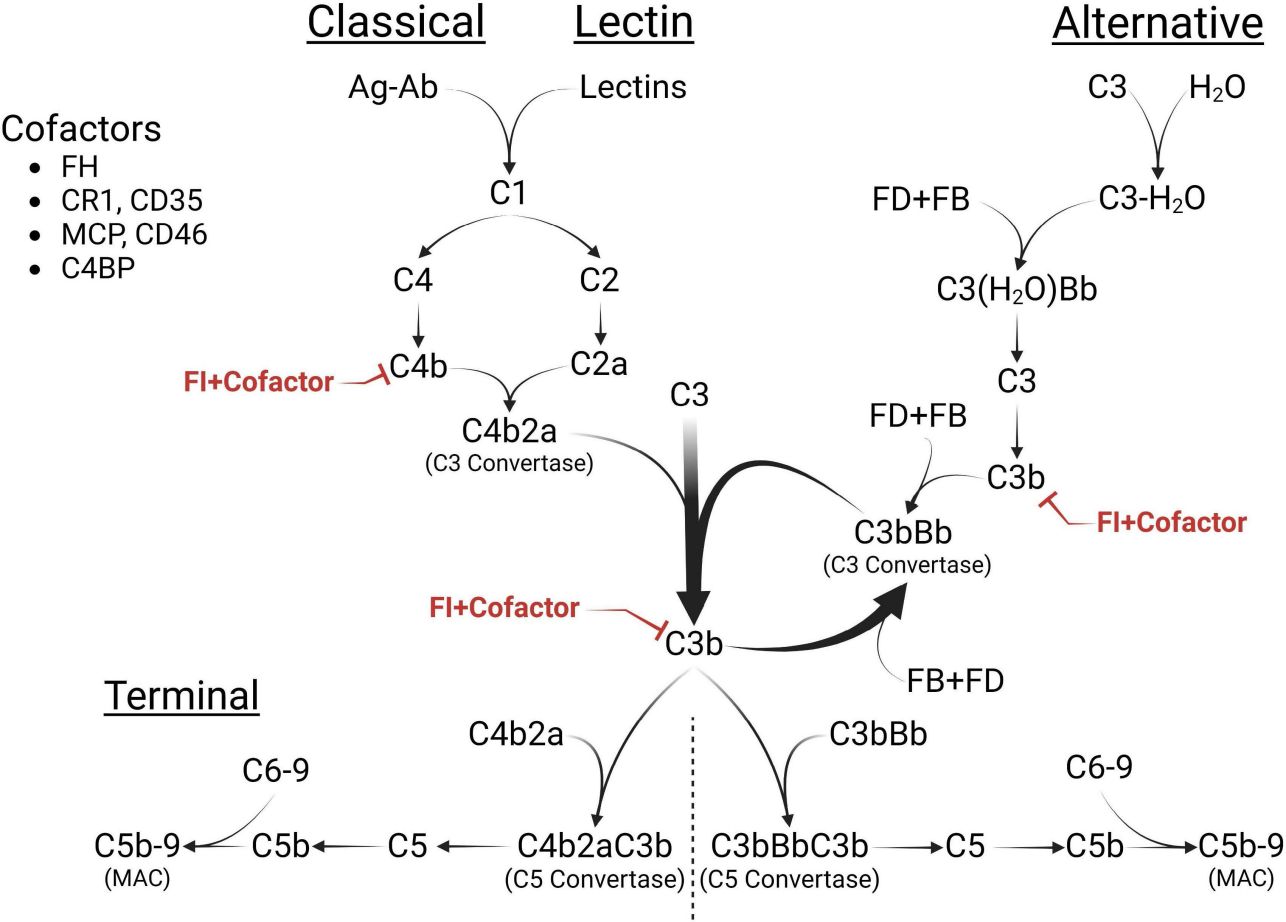
The Complement Cascade. The complement cascade is activated through three pathways: classical (CP), lectin (LP), and alternative pathways (AP). While the CP and LP are triggered by specific stimuli (immune complexes or lectins, respectively), the AP is continuously active, initiated through the spontaneous hydrolysis of C3 [C3(H_2_O)]. All pathways converge at C3 cleavage, forming the C3 convertases [C4b2a, C3(H_2_O)Bb or C3bBb]. C3(H_2_O) shares structural similarity with C3b and interacts with factor B (FB) and factor D (FD) to generate the AP initiating C3 convertase, C3(H_2_O)Bb. The resulting C3b can form more C3 convertases, C3bBb, thus amplifying the AP, or activate the terminal pathway (TP) by creating the C5 convertase (C3bBbC3b) of the AP or the C5 convertase (C4b2aC3b) of the CP. Both of these convertases lead to the formation of the membrane attack complex (MAC), C5b-9, on cell surfaces. FI and its cofactors [Factor H (FH), C4 binding protein (C4BP), membrane cofactor protein (MCP), or complement receptor 1 (CR1)] downregulate cascade activity by inactivating C4b and C3b and preventing the continued propagation of all pathways. Figure was created using BioRender.com.

The complement cascade is a cornerstone of innate immunity, marking infected and damaged cells for removal through opsonization, lysing cells via the membrane attack complex (MAC), and triggering the humoral immune response (1). Activation of the complement system occurs through three pathways: classical, lectin, and alternative. The classical pathway is initiated by antigen-antibody complexes, while the lectin pathway is triggered by lectin binding to mannose proteins (1). Both pathways generate the C3 convertase, C4b2a. In contrast, the alternative pathway is constitutively active due to the spontaneous hydrolysis of C3, leading to the formation of another kind of C3 convertase, C3(H_2_O)Bb, in a process known as tick-over (2, 3). Both C4b2a and C3(H_2_O)Bb subsequently lead to the formation of the C3 convertase, C3bBb (3). This complex, in turn, activates the terminal pathway, facilitating the production of the MAC, C5b-9 (**Figure 1**) (1, 2, 4).

The regulatory serine protease, complement Factor I (FI), together with its cofactors (FH [factor H], C4BP [C4 binding protein], MCP [membrane cofactor protein], and CR1 [complement receptor 1]), plays a crucial role in regulating complement activity by deactivating C4b and C3b (**Figure 1**) (5). Partial FI deficiency increases baseline complement activity and raises susceptibility to several complement-mediated diseases such as age-related macular degeneration (AMD), C3 glomerulopathy (C3G), and complement-mediated thrombotic angiopathy/atypical hemolytic uremic syndrome (complement-mediated TMA/aHUS) (6–10). Complete FI deficiency, on the other hand, leads to fluid-phase complement consumption and diseases such as severe myelitis, meningitis, and encephalitis (11–14). This phenotypic variability reflects the influence of other complement protein levels, such as FH in the fluid phase and MCP on cell membranes (5), further illustrating the complexity of the complement cascade.

Classifying rare missense variants in *CFI* is challenging due to inconsistencies between studies. For instance, the variant NM_000204.5:c.355G>A (p.Gly119Arg), is classified in ClinVar as “conflicting interpretations” as it has been documented as pathogenic (P), likely pathogenic (LP), variant of uncertain significance (VUS), and likely benign (LB) (15–25). These inconsistencies present significant challenges for patient care. It is important to note that genetic variants can substantially affect serum FI levels; our previous study estimated that up to 80% of rare variants (MAF < 0.1%) impact serum levels, categorizing them as type 1 variants (5).

In this study, we focused on *CFI* variants with minor allele frequencies less than 0.1% that were associated with low or low-normal plasma levels (below the first quartile of the normal reference range: 18-44 mg/L). For each variant, we conducted a minigene expression assay to determine the impact on splicing (26–28). We hypothesized that some rare variants disrupt normal splicing, resulting in functionally null alleles and haploinsufficiency for FI.

## Methods

### Variant nomenclature and identification

*CFI* variants were annotated with reference to the NCBI reference transcript NM_000204.5 following Human Genome Variation Society (HGVS) guidelines. To identify variants, a targeted sequencing panel was performed on an Illumina MiSeq (Illumina, California, USA), and the data were analyzed using a custom bioinformatic pipeline. Variants were filtered to include those with a Quality Depth (QD) ≥ 10, a variant quality score (Qvar) ≥ 50, MAF < 0.1%, and variants classified as non-synonymous, indels, or splice-site variants (5). Only *CFI* missense variants and those in splicing regions were included in this study. Ethical approval for this study was granted by the Ethics Committee of the University of Iowa.

### Patients

After identifying patients with ultra-rare variants in *CFI* (MAF < 0.1%), we measured FI levels using either an enzyme-linked immunosorbent assay (ELISA) or a radial immunodiffusion (RID) assay. For the ELISA, we used the MicroVue Factor I EIA kit (QuidelOrtho, CA) according to the manufacturer’s protocol. For the RID assay, we loaded patient samples and standards in wells on a pre-made agarose plate with an anti-factor I antibody (The Binding Site, Birmingham, UK). After a 72 hour incubation, diffusion rings were measured with an RID plate reader (The Binding Site, Birmingham, UK). If levels of FI were < 25 mg/L (first quartile of the reference range: 18-44 mg/L) by ELISA, and confirmed with RID results (normal reference range: 16-40 mg/L), the variant was considered for further study.

### Variant classification

We searched the ClinVar database (http://www.ncbi.nlm.nih.gov/clinvar/), PubMed, Franklin Genoox (https://franklin.genoox.com/clinical-db/home), and the complement database (https://www.complement-db.org/) to identify previously classified *CFI* variants. Variant frequencies were estimated using the Genome Aggregation Database (gnomAD, https://gnomad.broadinstitute.org/); classification followed ACMG guidelines (29, 30).

### *In silico* prediction

To analyze potential impacts on splicing, variants were evaluated using the splicing algorithms SpliceAI and Human Splice Finder (HSF) (31, 32). We used the bioinformatic tool ESEfinder to assess effects on *cis*-acting elements (33, 34). Comparative analyses were conducted to determine whether a missense change suppressed native *cis*-acting elements or introduced novel elements compared to the wild-type controls.

### Minigene assay

Primers were designed for the cloning of four groups of exons— 2-3, 4-6, 9-10, and 11 (**Supplementary Table 1**)—including at least 200bp of 5’ and 3’ intronic sequences and the intervening intronic sequences between exons (where appropriate) into the multi-cloning site of the pET01 construct (MoBiTec, Goettingen, Germany). Genomic DNA from patients served as the template for PCR amplification with a high-fidelity *Taq* DNA polymerase (Phusion 5, New England Biolabs). The pET01 vector was linearized using the restriction enzymes *BamHI* and *XhoI* (New England Biolabs) and treated with Quick CIP (New England Biolabs). PCR products were cloned into the pET01 vector using NEBuilder HiFi DNA Assembly Master Mix (New England Biolabs). Cloning was facilitated by including in each primer a 5’ overhang of 20 nucleotides complementary to the plasmid.

Positive colonies, both with rare variants and wild type, were identified through colony PCR followed by Sanger sequencing. Plasmid constructs were harvested after amplification and purification using the QIAprep Spin Miniprep Kit (Qiagen). Transfection into HEK-293 (ATCC CRL-1573, human embryonic kidney) and Hep-G2 cell lines (ATCC HB-8065, human liver hepatocellular carcinoma) was carried out using Lipofectamine LTX + PLUS Reagent (ThermoFisher Scientific).

Cells were harvested 48 hours post-transfection, and RNA isolation was performed using the RNeasy Plus Mini Kit (Qiagen). Subsequently, 500 ng of RNA was reverse transcribed using Oligo d(T) primers and SuperScript IV Reverse Transcriptase (ThermoFisher Scientific). The synthesized cDNA was then amplified by PCR using plasmid-specific primers. The resulting products were resolved by electrophoresis on a 1% or 1.5% agarose gel at 100V for two hours, followed by staining with SYBR green dye (Invitrogen). Bands of interest were extracted from the gels using the QIAquick gel extraction kit (Qiagen) and subjected to Sanger sequencing.

## Results

### Variants and patients

**Figure 2:**
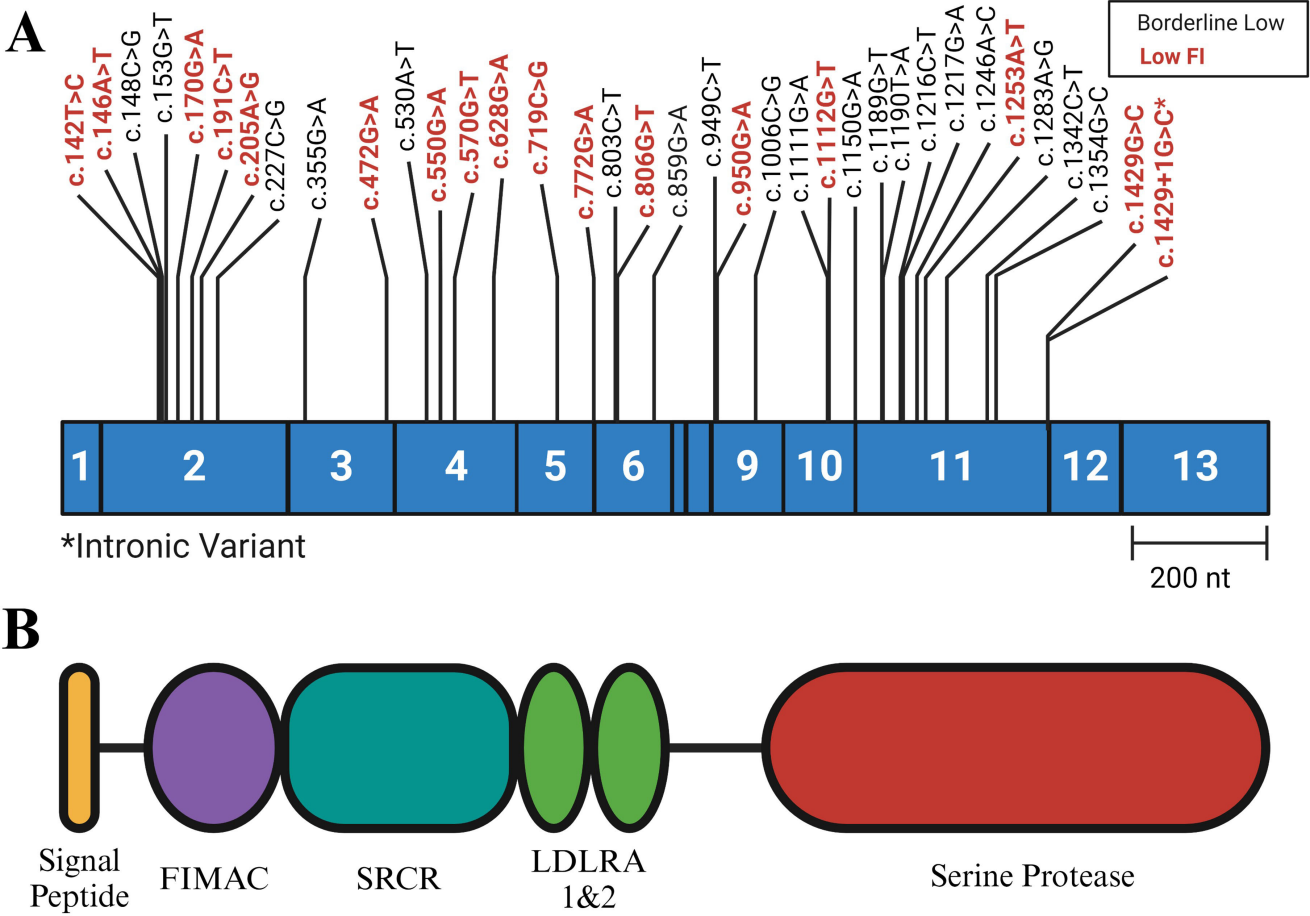
Variant Positions in Complement Factor (I) **(A)** Exonic representation of variants studied. The variants shown in black are associated with low normal levels of FI (< 25 mg/L, normal reference range 18-44 mg/L), while the variants labeled in red are associated with low FI (< 18 mg/L). **(B)** FI protein structure. The heavy chain includes the FI Membrane Attack Complex (FIMAC), Scavenger Receptor Cysteine Rich (SRCR), and the two Low Density Lipoprotein (LDLRA1 and LDLRA2) domains. The light chain consists of the Serine Protease (SP) domain. Each domain is encoded by the exonic region shown directly above. NCBI RefSeq: NM_000204. Figure was created using BioRender.com.

Thirty-six of 120 rare *CFI* variants were selected for further study based on FI expression levels (**Figure 2**). These variants were identified in 56 of 2,262 patients diagnosed with complement-mediated TMA/aHUS, Shiga toxin-producing *Escherichia coli*-associated hemolytic uremic syndrome (STEC-HUS), C3G, or recurrent infections. The 36 variants included 35 missense variants and 1 splice site variant (**Figure 2**; **Supplementary Tables 2, 3**); disease phenotypes in the 56 patients included complement-mediated TMA/aHUS (35 patients), STEC-HUS (1 patient), C3G (10 patients), both C3G and complement-mediated TMA/aHUS (1 patient), and recurrent infections (2 patients). A definitive diagnosis was not available for 7 patients (**Supplementary Table 2**).

**Figure 3:**
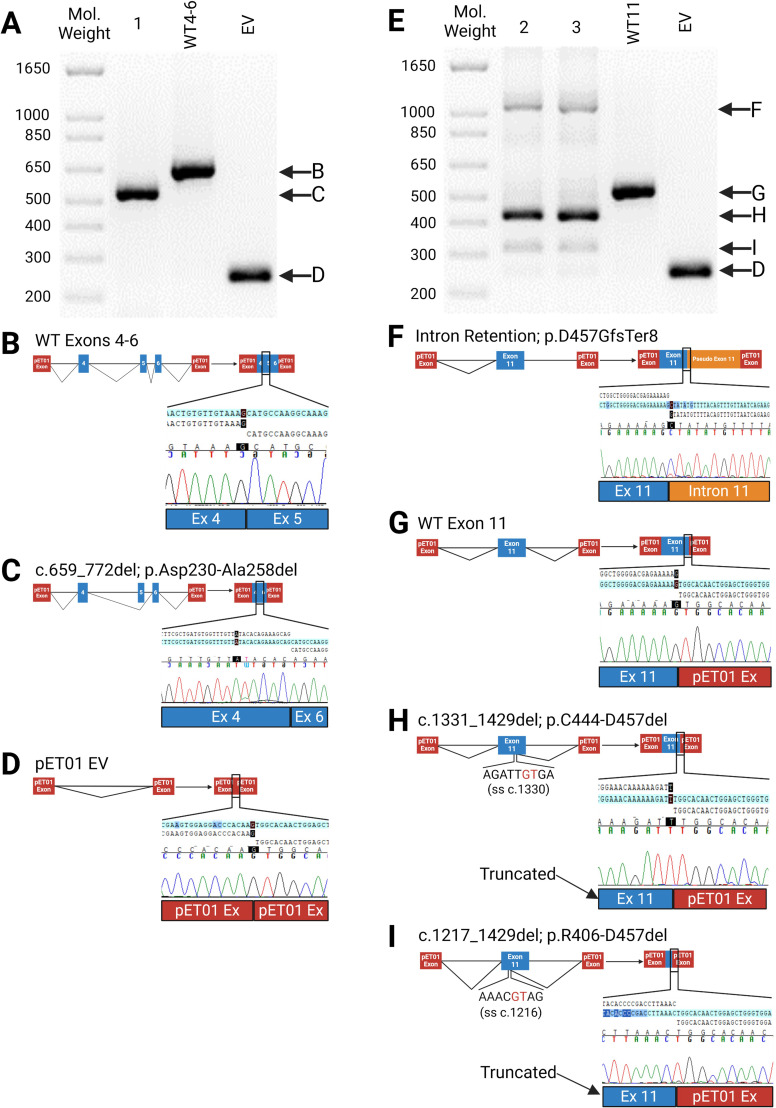
Impacts of Splice Site (SS) Variants. **(A–D)** Results of minigene assay (RT-PCR) with Sanger sequencing confirmation for c.772G>A (Sample 1) and wildtype construct (WT4-6); **(B)** normal splicing of exons 4-6 with corresponding Sanger sequencing results indicating the boundary between exons 4 & 5; **(C)** Sanger sequencing confirmation of Exon 5 skipping, which leads to an in-frame deletion in the mature mRNA (c.659-772del; p.Asp230-Ala258del); **(D)** Empty pET01 Vector (EV); **(E–I)** Splicing effects of c.1429+1G>C (sample 2), c.1429G>C (sample 3) and wildtype (WT11); **(F)** Intron 11 retention in the mRNA (p.Asp457GlyfsTer8); **(G)** normal splicing; **(H, I)** Aberrant splice utilizing a cryptic splice site in exon 11, resulting in two in-frame deletions: c.1331_1429del (p.Cys444-Asp457del) and c.1217_1429del (p.Arg406-Asp457del). **(A, E)**, 1.5% agarose gel. Figure was created using BioRender.com.

**Figure 4:**
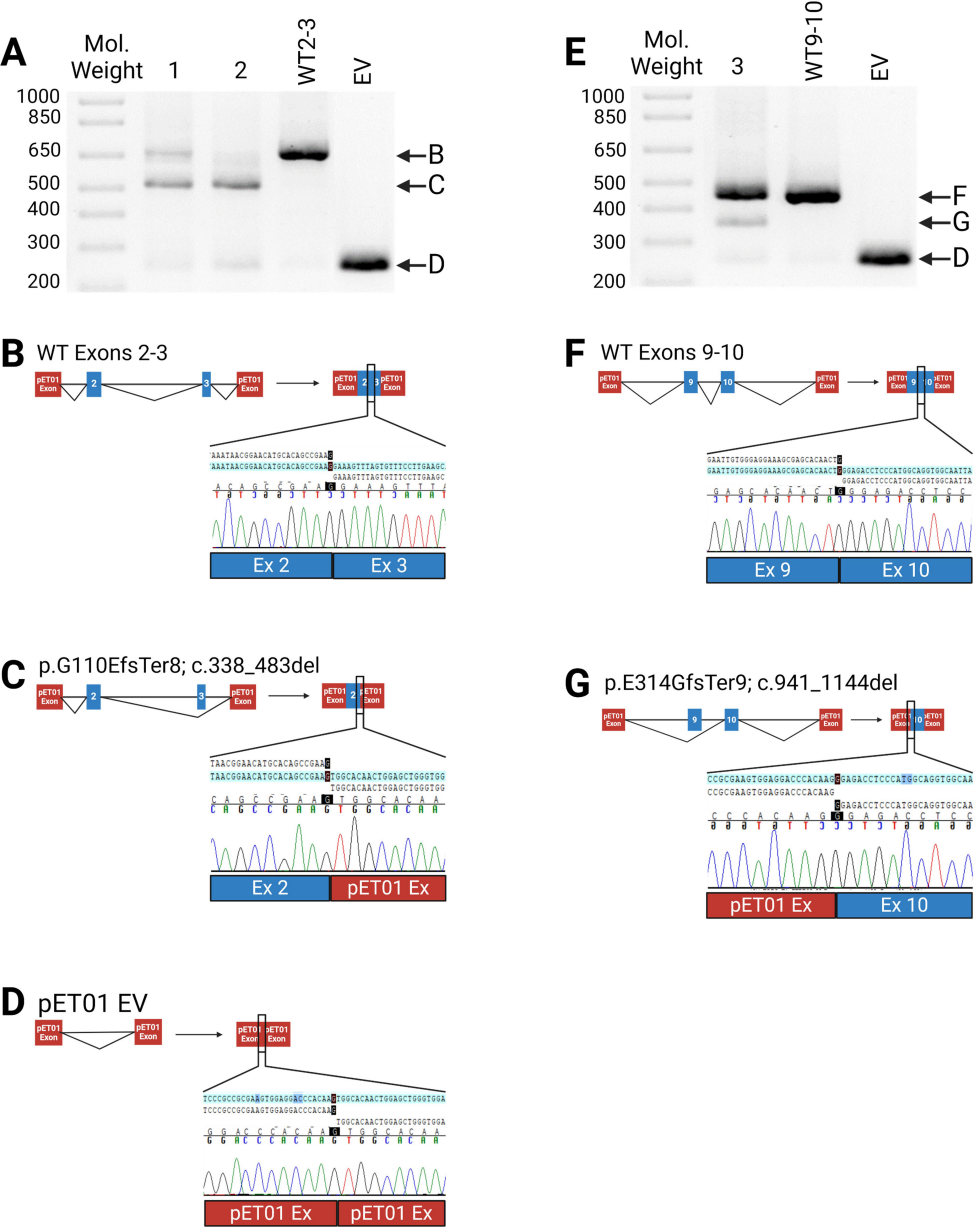
Impact of Exonic Variants. **(A–D)** Results of minigene assay (RT-PCR) with Sanger sequencing confirmation for c.472G>A (sample 1), c.355G>A (sample 2) and the wildtype (WT2-3); **(B)** Normal splicing with Sanger sequencing indicating the exons 2-3 boundary; **(C)** Aberrant splicing leading to an out-of-frame deletion of exon 3. Sanger sequencing confirmed exon 3 skipping; **(D)** Empty pET01 vector (EV); **(E–G)** effects of c.950G>A (sample 3) and wildtype (WT9-10); **(F)** Normal splicing showing the exon 9-10 boundary; **(G)** Aberrant splicing leading to an out-of-frame deletion of exon 9. **(A, E)**, 1.5% agarose gel. Figure was created using BioRender.com.

Six of 36 variants (NM_000204.5:c.335G>A, p.Gly119Arg; c.472G>A, p.Gly158Arg; c.772G>A, p.Ala258Thr; c.950G>A, p.Arg317Gln; c.1429G>C, p.Asp477His; and c.1429+1G>C) led to aberrant splicing in both the HEK-293 and Hep-G2 cell lines (**Figures 3**, **4**). The remaining 30 variants had no impact on transcription (**Supplementary Table 3**). While the outcome for c.772G>A (p.Ala258Thr) has been documented (35) and c.1429+1G>C can be predicted to affect the canonical splicing site, we used ACMG criteria to reclassify the remaining variants from VUS to LP (c.472G>A, p.Gly158Arg and c.950G>A, p.Arg317Gln) and P (c.355G>A, p.Gly119Arg and c.1429G>C, p.Asp477His) (**Table 1**).

**Table 1:** Variant classification.

Variant	Protein	ClinVar Classification^±^	Franklin Classification^±^	New Evidence of pathogenicity	Classification
c.355G>A	p.Gly119Arg	Conflicting interpretations	LP	PVS1_very strong, PM2*, PP4	P
c.472G>A	p.Gly158Arg	Not reported	VUS	PVS1_strong, PM6, PM2, PP4	LP
c.950G>A	p.Arg317Gln	VUS	VUS	PS3_RNA, PM2, PP4	LP
c.1429G>C	p.Asp477His	VUS	VUS	PVS1, PM2, PM1, PP4	P

^±^Classifications as of 04/2024.

*gnomAD v3.1.2 (GRCh37/hg19; ENSG00000205403.8).

### Computational prediction

SpliceAI and Human Splice Finder (HSF) provide a range of possibilities for predictive impacts on splicing. For SpliceAI, a splice score > 0.70 is considered to have a strong impact on splicing, scores between 0.22 and 0.70 are considered to have a possible impact on splicing, and scores < 0.22 are considered to have no impact on splicing (36). For HSF, variants with an exon splicing enhancer to exon splicing silencer (ESE/ESS) motif ratio < (–4) are considered to impact splicing, while scores greater than that cutoff are not predicted to affect splicing.

With respect to SpliceAI, three variants—c.772G>A (p.Ala258Thr), c.1429G>C (p.Asp477His), and c.1429+1G>C—were predicted to impact splicing (splice score > 0.7) for a positive predictive value (PPV) of 50%. However, if variants with a moderate impact on splicing are included (0.22-0.70), then SpliceAI accurately predicted the splicing outcome for all positive variants—c.355G>A (p.Gly119Arg), c.472G>A (p.Gly158Arg), c.950G>A (p.Arg317Gln), c.772G>A (p.Ala258Thr), c.1429G>C (p.Asp477His), and c.1429+1G>C. Additionally, SpliceAI had a negative predictive value (NPV) of 100% as the software accurately predicted that neutral variants have no impact on splicing (splice score < 0.22).

With respect to HSF, the software strongly predicted the splicing outcome for three variants—c.772G>A (p.Ala258Thr), c.1429G>C (p.Asp477His), and c.1429+1G>C—yielding a positive predictive value (PPV) of 50%. However, if the cutoffs for HSF described previously are used, then the software accurately predicted the splicing outcome for all positive variants and had a PPV of 100%. Additionally, HSF had an NPV of 50% as the software correctly predicted the splicing outcomes for 15 of the neutral variants while incorrectly predicting the outcomes for the remaining 15 variants. The splice scores for the positive variants are summarized in **Table 2**, and the splice scores for all variants are summarized in **Supplementary Table 3**.

**Table 2:** Variant in-silico prediction and FI expression.

Variant	Protein	MAF^±^	Number of Pts in Cohort	Average FI expression (mg/L)	SpliceAI*	Human Splice Finder	Observed Effect
c.355G>A	p.Gly119Arg	0.04%	7	22.9	0.41	ESE/ESS (-4)	Exon 3 Skipping
c.472G>A	p.Gly158Arg	0.001%	1	10.2	(-0.01) & 0.30	Potential Impact	Exon 3 Skipping
c.719C>A	p.Ala240Gly	0.02%	7	20.7	(-0.07)	ESE/ESS (-11)	No Effect
c.772G>A	p.Ala258Thr	0.01%	3	10.9	(-0.73) & 0.04	Impact on Splicing	Exon 5 Skipping
c.949C>T	p.Arg317Trp	0.002%	1	Not Available	(-0.18)	No Impact	No Effect
c.950G>A	p.Arg317Gln	0.002%	2	21.3	(-0.36)	Potential Impact	Exon 9 Skipping
c.1429G>C	p.Asp477His	0.00002%	3	20.9	(-0.75) & 0.37	Impact on Splicing	Three Isoforms
c.1429+1G>C		0.00003%	2 (1 hom)	Undetectable in homozygous state	(-0.77) & 0.41	Impact on Splicing	Three Isoforms

^±^Collected from the gnomAD database (GRCh37/hg19; ENSG00000205403.8).

*Positive values indicate predicted splice gains, while negative values indicate predicted splice losses. Scores are from 0 to ±1, with 0 meaning no impact on splicing and ±1 meaning impact on splicing.

All patients are heterozygous except for the one labeled as hom (homozygous).

### Canonical splicing site variants

c.772G>A (p.Ala258Thr), which impacts the last nucleotide of exon 5 and has been documented to result in exon 5 skipping (35), was identified in three patients in our cohort. We confirmed its impact on exon 5 skipping and used it as a positive control in the minigene splicing assay (**Figures 3A–C**).

c.1429G>C (p.Asp477His) and c.1429+1G>C impact the last nucleotide of exon 11 and the first nucleotide of intron 11. While the variant, c.1429+1G>C, is pathogenic, the outcome of the adjacent nucleotide, c.1429G>C, is unknown (37, 38). Three patients in our cohort carry c.1429G>C (p.Asp477His) while two have the c.1429+1G>C variant. Both variants lead to three aberrant transcripts. The most common transcript uses a 5’ cryptic splice site within exon 11 that results in an in-frame deletion of 14 amino acids (c.1331_1429del, p.Cys444-Asp457del) (**Figure 3H**). Next is a transcript reflecting intron 11 retention and premature truncation (p.Asp457GlyfsTer8) (**Figure 3F**). The least abundant transcript results from another in-frame deletion of 64 amino acids (c.1217_1429del, p.Arg406-Asp457del) (**Figure 3I**).

### Exonic rare variants

Both variants in exon 3—c.335G>A (p.Gly119Arg) and c.472G>A (p.Gly158Arg)—lead to exon 3 skipping (**Figures 4A–C**). The c.355G>A variant was seen in five patients while the variant, c.472G>A, was seen in one patient in our cohort. Similarly, the variant associated with exon 9, c.950G>A, which was seen in two patients, causes aberrant splicing through the removal of exon 9 in a portion of its transcribed product (**Figures 4E–G**). The splice-altering variants in exons 3 and 9 result in an out-of-frame deletion of their respective exons (c.338_483del, p.G110EfsTer8 & c.941_1144del, p.E214GfsTer9).

### Assessing potential splicing mechanisms

**Figure 5:**
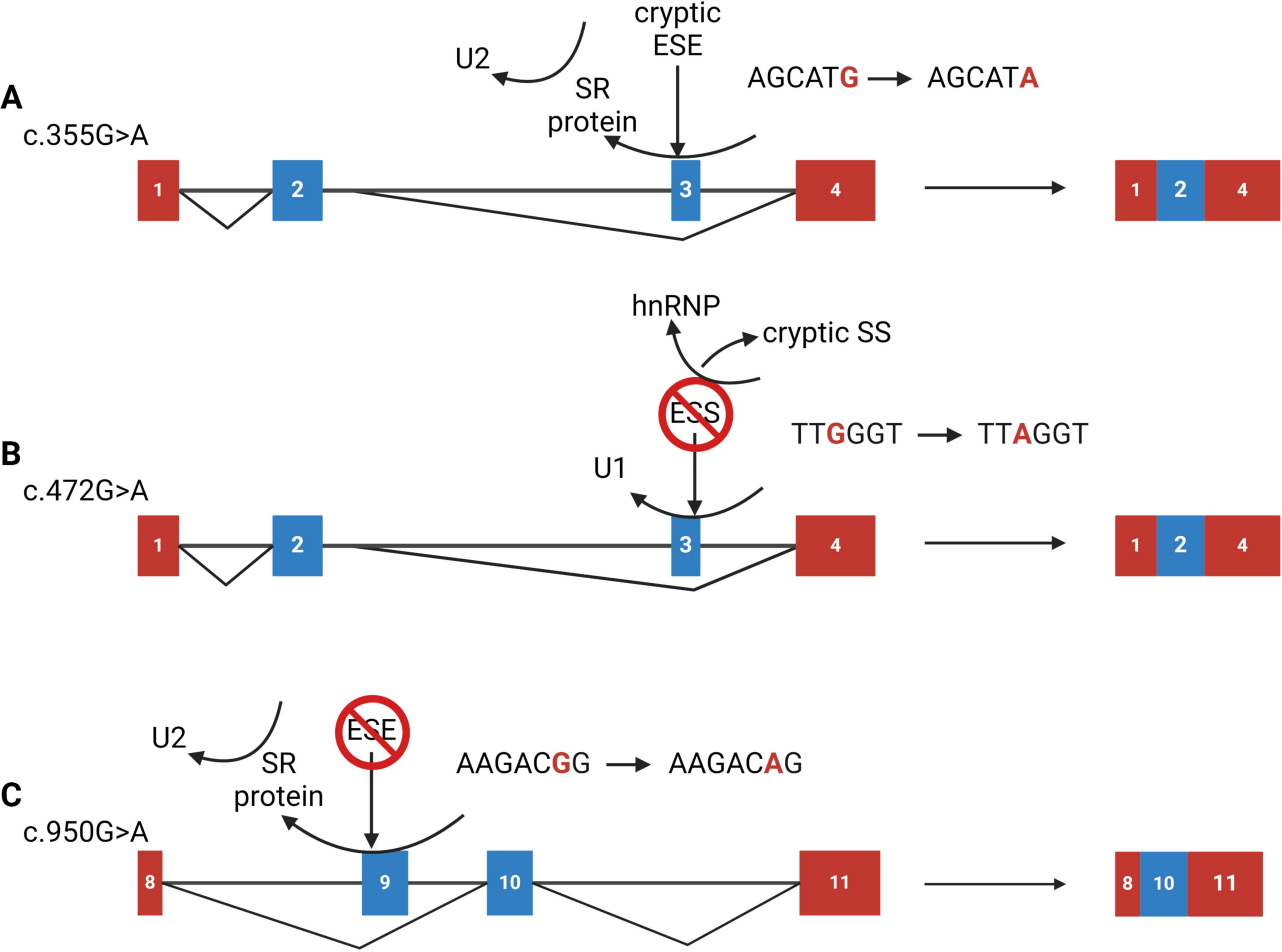
Proposed mechanisms for exonic variants that cause splicing defects. **(A)** The c.355G>A (p.Gly119Arg) variant is predicted to form a cryptic exonic splicing enhancer (ESE) site. This ESE site may compete with the native enhancer element, preventing the binding of the *trans-*acting element thus failing to recruit the spliceosome, resulting in exon 3 skipping; **(B)** The c.472G>A (p.Gly158Arg) variant is predicted to lose an exonic splicing silencer (ESS) site, potentially uncovering a cryptic splice site located upstream of the native 5’ splice site (SS). The cryptic SS may be competing with the native one and preventing the U1 snRNA molecule from demarking the 5’ SS resulting in exon 3 skipping; **(C)** The c.950G>A (p.Arg317Gln) variant is predicted to eliminate a native ESE site. The loss of this site impedes recognition from an SR-protein and thus recruitment of the U2 snRNA molecules leading to complete exon 9 skipping. Figure was created using BioRender.com.

In addition to predicting splicing outcomes, ESEfinder was used to identify whether the exonic variants were associated with *cis*-acting elements. ESEfinder predicted an impact for nearly all the variants tested (69%), suggesting inaccurate results. c.355G>A (p.Gly119Arg) is predicted both to create a cryptic ESE element and lose an ESS element; c.472G>A (p.Gly158Arg) is predicted to create a cryptic ESE while both losing and gaining an ESS; and c.950G>A (p.Thr317Gln) is predicted to lose an ESE site (**Figure 5**).

## Discussion

In this study, we investigated the potential correlation between rare *CFI* variants and abnormal RNA splicing and found that of 36 variants studied, six resulted in aberrant splicing: c.355G>A (p.Gly119Arg); c.472G>A (p.Gly158Arg); c.950G>A (p.Thr317Gln); c.772G>A (p.Ala258Thr); c.1429G>C (p.Asp477His); and c.1429+1G>C. While the consequence of c.772G>A (p.Ala258Thr) has been previously documented (35, 39), this study is the first to detail the functional impact of the remaining five variants, including c.1429+1G>C. Although c.1429+1G>C has been reported previously (37, 38), its consequence has never been demonstrated.

Correct RNA splicing is facilitated by the unambiguous demarcation of exon-intron boundaries, which the spliceosome recognizes by the presence of canonical sequences: the 3’ and 5’ splice sites (SS); the branch point (BP) with its crucial internal adenine; and the polypyrimidine-tract complex (PPT) near the 3’ acceptor site (40). In addition, *trans*-acting factors such as serine-arginine (SR) proteins and heterogeneous ribonuclear proteins (hnRNPs) regulate splicing. The former promotes splicing by binding to enhancer sequences and aiding spliceosome recruitment, while the latter inhibit splicing at silencer sequences. Splicing machinery relies on these factors and their corresponding *cis*-acting elements—exon/intron splicing enhancers/silencers (ESE, ESS, ISE, ISS)—for efficient splicing (34, 40–42). However, the presence of canonical sequences alone does not guarantee proper splicing, as demonstrated by c.355G>A (p.Gly119Arg), c.472G>A (p.Gly158Arg), and c.950G>A (p.Arg317Gln). These three exonic variants maintain the canonical splicing sequences but disrupt *cis*-acting elements, illustrating that for correct splicing to occur, consensus sequences and intact *cis*-acting elements, along with their associated *trans*-acting elements, are essential.

The splice site variants—c.772G>A (p.Ala258Thr), c.1429G>C (p.Asp477His), and c.1429+1G>C—alter the 5’ donor canonical sequence. As a result, the U1 snRNA molecule fails to recognize the native 5’ splice site, prompting it to scan upstream for the next suitable donor site. With respect to c.772G>A (p.Ala258Thr), the next suitable splice site is the splice donor of exon 4, which results in an in-frame deletion of exon 5 (p.Asp230-Ala258del). For c.1429G>C (p.Asp477His) and c.1429+1G>C, the next suitable splice site is embedded within exon 11, the consequence of which is an in-frame deletion (p.Cys444-Asp457del). While it was expected that the splice site variant c.1429+1G>C would disrupt splicing, the impact of c.1429G>C, (p.Asp477His) on transcription was not recognized (37).

To assess the impact of the in-frame deletions caused by c.772G>A (p.Ala258Thr), c.1429G>C (p.Asp477His), and c.1429+1G>C, we used Alphafold2, which predicted disruption of the geometry of the serine protease's active site (43). c.772G>A (p.Ala258Thr) results in the deletion of p.Asp230-Ala258, which eliminates most of the LDLRA1 domain. These structural alterations likely trigger proteolysis in the endoplasmic reticulum and result in haploinsufficiency of FI (**Table 2**).

The exonic variants—c.355G>A (p.Gly119Arg), c.472G>A (p.Gly158Arg), and c.950G>A (p.Arg317Gln)—disrupt regulatory elements that enhance splicing. While the c.335G>A results in complete exon skipping, the effects of c.472G>A and c.950G>A are partial, leading to a mixture of normal and aberrant splicing (**Figure 4**). Using ESEfinder (33, 34), we have proposed mechanisms for how these variants disrupt splicing (**Figure 5**). For instance, c.355G>A gains an ESE element and alters the hnRNP recognition sequence. The addition of the cryptic ESE may impede the binding of the native SR-protein and lead to failure of U2 recruitment, resulting in exon 3 skipping. The variant c.472G>A gains an ESE *cis*-acting element, uncovers a cryptic 3’ acceptor site closely upstream of the native 5’ donor site, and causes a loss of an ESS *cis*-acting element. The loss of the ESS and the addition of the ESE may strengthen the cryptic splice site and result in improper U1 binding and ultimately exon 3 skipping. Finally, c.950G>A destroys a native ESE *cis*-acting element, potentially perturbing recruitment of splicing machinery to the acceptor site, which in some transcripts leads to skipping of exon 9, however, the majority of the transcripts remain unaffected (**Figure 4E**). Notably, the splicing pattern for c.949C>T (p.Arg317Trp), located one nucleotide upstream, is unaffected. While ESEfinder offers insights into potential splicing impacts, all three exonic variants result in out-of-frame deletions for exons 3 and 9 (p.Gly110GlufsTer8 and p.Glu314GlyfsTer9, respectively).

HSF and ESEfinder have important limitations. By way of example, c.719C>G (p.Ala240Gly) is strongly predicted to impact splicing by HSF with an ESE/ESS motif ratio of -11, and ESEfinder predicts a loss of a native *cis*-acting element. However, our minigene assay showed no effect on splicing, suggesting the reason for haploinsufficiency is unlikely to be at the transcriptional level. Previous studies have associated the c.719G>C (p.Ala240Gly) variant, which resides in the LDLRA1 calcium-binding domain, with disruption of proper calcium binding, which leads to improper folding and premature protease degradation (8). Consistent with this proposed mechanism, other substitutions at p.Ala240 disrupt folding of the heavy chain of FI (44). Similarly, substitutions at p.Arg317, p.Lys267 and p.Gly287 lead to decreased FI secretion or enzymatic activity (8, 17, 44, 45). These findings underscore the complexity in evaluating variant effect on FI expression.

Understanding the correlation between *CFI* variant effects and FI expression is vital, and while doing so by ELISAs is practical, the accuracy of some kits is compromised by the presence of a common SNP (*CFI* c.1217G>A, p.Arg406His) with a minor allele frequency of 11% in East Asians. The presence of this SNP does not disrupt splicing, rather, it inhibits binding between FI and the capture antibody, which results in falsely low measurements by ELISA (**Supplementary Table 4**). Thus, the availability of alternative methodologies, such as the radial immunodiffusion assays (RID), is advisable if this SNP is detected. When multiple methods show consistently low levels of FI expression and a *CFI* variant is found, further investigation, such as a minigene assay or protein functional testing should be conducted to determine whether the variant has any pathogenic effects. If the minigene assay is impractical *in silico* splicing predictors like SpliceAI can be used to address the potential correlation between FI levels and pre-mRNA splicing.

In summary, we have demonstrated how the minigene splicing assay can be used to assess the impact of variants on splicing by identifying 4 novel splice-altering variants, which we have reclassified as likely pathogenic [c.472G>A (p.Gly158Arg) and c.950G>A (p.Arg317Gln)] or pathogenic [c.355G>A (p.Gly119Arg) and c.1429G>C (p.Asp477His)] following the American College of Medical Genetics and Genomics (ACMG) criteria (**Table 1**) (29, 30). Additionally, we have accounted for alternative rationales for haploinsufficiency for FI through improper folding as exemplified in the variants c.772G>A (p.Ala258Thr), c.1429G>C (p.Asp477His), and c.1429+1G>C. Overall, we have offered alternative methods for classifying ultra-rare variants beyond those found in *CFI*, utilizing both the ACMG guidelines and the minigene splicing assay.

## Data Availability

The original contributions presented in the study are included in the article/**Supplementary Material**. Further inquiries can be directed to the corresponding authors.
