# Supplementary material for: Functional evaluation of rare variants in complement factor I using a minigene assay
Source: Front Immunol. 2024 Aug 22;15:1446081. doi: 10.3389/fimmu.2024.1446081 (PMC11374653; doi:10.3389/fimmu.2024.1446081)
Supplement: Supplementary file 1 [file Datasheet1.pdf]

frontiers

Supplementary Material

Supplementary Table 1. **Primer Sequences*.***

Purpose	Forward	Reverse
Exons 2-3	TAGCCAGTTTTCCCAGCACC	TGCTTTGTTGTCAGCAGGGT
Exons 4-6	CCACCCCCAGCCAAGTTTTA	TGTATGTGCAGTGAGACCGT
Exons 9-10	GCCAGCCCTTTTCTGCCA	ATAGGTAGGCCACAGTGGAG
Exon 11	ATGCTGTCTGGTCTCTGCTT	AGCAATAAATCAAGCCTCA
Exon 12	AGGGACTGTGAGAAGATAGGAGGTA	GGACAAATTATGTAACTGCTCTGCA
Colony PCR	ACATGGTAGCTGCCAGGAAG	AAGGGTTGCATGGCTGTGAG
Sequencing Colony PCR	GTAGCTGCCAGGAAGGAGTG	GGCCTCCAAAACCTACACAT
cDNA Amplification	GATGGATCCGCTTCCTGCCC	CCTCCGGGCCACCTCCAGTGCC
Sequencing Amplified cDNA	GGATTCTTCTACACACCC	TCCACCCAGCTCCAGTTG

Above the dotted line are the primers used to amplify *CFI* regions from patient DNA, while below the dotted line are the primers utilized for verification. The sequence ATAGGTACCGGGCCCCCCCTCGAG was appended to the 5’ end of the forward insert primers, and the sequence GGCCGCTCTAGAACTAGTGGATCC was added to the 5’ end of the reverse insert primers.

Supplementary Table 2. **Demographic and Biomarker Profile in Patients with *CFI* Variants**

ID	CFI Variant	Zygosity	Other Complement Variants	Sex	Diagnosis	CH50	APFA	C3 (g/L)	C4 (g/L)	C5 (mg/L)	P (mg/L)	Bb (mg/L)	C5b-9 (mg/L)	FI (mg/L)	FH (mg/L)
1	c.142T>C; c.146A>T	Het, on same allele		M	aHUS	Normal	76%	1.2	0.52	25.9	16.3	2.3	0.3	13.1	388
2	c.148C>G	Het		F	aHUS	Normal	60%	1	0.32	18.3	13.6	0.8	0.1	15.2	235
3	c.148C>G	Het		M	HUS	Low	95%	1.1	0.28	26	12.3	9.2	0.8	27.3	256
4	c.148C>G	Het		F	C3G										
5	c.153G>T	Het	Del (*CFHR3-CFHR1*) het	F	aHUS	Absent	9%		0.21	41.1	11.7		0.12	11.9	221
6	c.170G>A	Het		F	C3G	Normal	12%	1.1	0.28	21.6	12.6	1.7	0.41	16.1	305
7	c.191C>T	Het	*CD46* c.191G>A het (P) *CFH* c.3553G>C het (VUS)	F	aHUS										
8	c.191C>T	Het		F	aHUS										
9	c.191C>T; c.205A>G	Compound Het	Del (*CFHR3-CFH1*) het	F	aHUS										
10	c.355G>A	Het		F	aHUS	Normal	110%	1.1	0.52	27.7	17.1		0.8	22.1	224
11	c.355G>A	Het		F	aHUS	Absent	0%	1.5	0.55	39.7	13.2			23.7	314
12	c.355G>A	Het	Del (*CFHR3-CFH1*) het	F	aHUS										
13	c.355G>A	Het	*C3* c.4594C>T het (VUS) *C6* c.542C>T het (VUS)	F	C3G										
14	c.355G>A; 859G>A	Compound Het		F	aHUS	Absent	10%	0.7	0.27	23	18.7	3.6	0.23	7.8	257
15	c.355G>A	Het	*CFH* c.3628C>T het (P)	M	C3G	Low	54%	1	0.37	20.4	14.4	3	0.27	16.6	304
16	c.355G>A	Het	*CD46* c.565T>G het (VUS)	F	aHUS										
17	c.472G>A; c.1246A>C	Compound Het		M	aHUS	Normal		0.8	0.36	18	11.6		0.48	10.2	267
18	c.530A>T	Het	Del (*CFHR3-CFH1*) het	F	aHUS										
19	c.550G>A	Het		F	aHUS	Normal	86%	1.2	0.29	22.9	16.1	3.1	1	17.6	279
20	c.570G>T	Het	Del (*CFHR3-CFH1*) het	F	NA*									16.6	223
21	c.570G>T	Het		M	C3G/ aHUS		0%	1	0.35	34.4	9.3	1.1	0.35	7.2	235
22	c.628G>A	Hom	Del (*CFHR3-CFH1*) hom	F	aHUS	Low	47%	0.6	0.12	11	9.4	3.4	0.08	14	163
23	c.628G>A	Het	Del(*CFHR3-CFH1*) hom	M	aHUS										
24	c.719C>G	Het		F	aHUS										
25	c.719C>G	Het	*CFH* c.790+1G>A het (P)	M	C3G	Low	0%	1	0.44	38.2	24.7	2.3	0.17	18.8	141
26	c.719C>G	Het	Del (*CFHR3-CFH1*) het	F	aHUS										
27	c.719C>G	Het		M	aHUS		75%	1.6	0.47				0.15	20.7	320
28	c.719C>G	Het	*C3* c.2203C>T het (LB)	F	C3G	Normal	54%	1.3	0.23	18.6	15.9	1.3	0.22	22.6	329
29	c.772G>A	Het		F	aHUS			0.8	0.25			1.8		10.8	242
30	c.772G>A	Het		M	aHUS										
31	c.772G>A	Het		F	aHUS										
32	c.803C>T	Het		F	aHUS			1	0.46	20.8	15.2	5.9	0.18	15.3	363
33	c.806G>T	Hom		M	RI**	Low	0%	0.6	0.27	10.9	5.2	4.8	0.32	<3	206
34	c.859C>A	Het		F	aHUS	Absent	10%	0.7	0.27	23	18.7	3.6	0.23	7.8	257
35	c.859C>A	Het		F	aHUS	Normal	76%	0.6	0.22	15.4	14	2	0.47	22.3	237
36	c.859C>A	Het		M	NA*									20.3	280
37	c.949C>T	Het		F	aHUS										

38	c.950G>A	Het		M	aHUS	Absent	0%	1	0.21	26.3	17.2	2	0.22	17.8	224
39	c.950G>A	Het		F	NA*										
40	c.1111G>A	Het		M	C3G	Normal	73%	1.7	0.55	19.2	16.9	2	0.23	20.3	374
41	c.1112G>T	Het		F	aHUS	Normal	94%	0.7	0.37	21.6	16.8	3.2	0.25	17.1	215
42	c.1150G>A	Het		M	aHUS										
43	c.1189G>T	Het	*CFH* c.575G>A het (VUS)	F	aHUS										
44	c.1190T>A	Het		M	aHUS	Normal	121%	0.9	0.46	18.3	15	0.95	0.2	20.4	210
45	c.1216C>T	Het		F	NA*							1.1	0.14	15.9	
46	c.1246A>C	Het		F	NA*										
47	c.1253A>T	Het	Del (*CFHR3-CFHR1*) hom	M	NA*		0.12%						0.75	11.7	
48	c.1283A>G	Het	*CFH* c.1056T>A het (P)	F	C3G	Normal	82%	1.1	0.43	21	11.5	5.4	0.43	23.7	184
49	c.1342C>T	Het	Del (*CFHR3-CFH1*) het	F	aHUS	Normal		1.6	0.4	30.8	17.8			20.2	394
50	c.1354G>C	Het		F	aHUS										
51	c.1354G>A	Het		F	aHUS										
52	c.1429G>C	Het	Del (*CFHR3-CFH1*) het	F	NA*										
53	c.1429G>C	Het		M	C3G			1.7	0.41		13.9		0.2	26	
54	c.1429G>C	Het		F	aHUS	Normal	59%	1.2	0.17	26	12.9	1.9	0.45	15.8	340
55	c.1429+1G>C	Het		M	C3G										
56	c.1429+1G>C	Hom	Del (*CFHR3-CFH1*) het	F	RI**	Low	0%	0.3	0.32	6.5	4.3	10	2.15	0	141

Reference ranges: APFA (50%-130%); C3 (0.9-1.8 g/L); C4 (0.15-0.57 g/L); C5 (13.5-27.0 mg/L); Properdin (10-33 mg/L); sC5b-9 (<0.3 mg/L); FI (18-44 mg/L); FH (180-420 mg/L)

* = Not Available;

** = recurrent infection;

Supplementary Table 3. **Variant Information and In Silico Predictions.**

Variant ID	Variant	Protein	MAF*	ClinVar Classification	Franklin Classification	SpliceAI Prediction#	HSF$ Prediction	ESEfinder	Observed Effect
4-110687896-A-G	c.142T>C	p.Cys48Arg	Novel	VUS	VUS	0.02	No Impact	Loss ESE	NES**
4-110687892-T-A	c.146A>T	p.Gln49Leu	Novel	VUS	VUS	(-0.02)	Potential Splicing Alterations: activation of cryptic acceptor site	Loss ESE	NES**
4-110687890-G-C	c.148C>G	p.Pro50Ala	9.55E-05	VUS	VUS	(-0.03) & 0.01	Significant Alteration of ESE/ESS motif ratio (-5)	Gain ESS	NES**
4-110687885-C-A	c.153G>T	p.Trp51Cys	6.57E-06	Not Reported	VUS	(-0.01)	Significant Alteration of ESE/ESS motif ratio (-2)	Gain ESE	NES**
4-110687868-C-T	c.170G>A	p.Gly57Asp	3.98E-06	Not Reported	VUS	0.04	No Impact	Gain ESE	NES**
4-110687847-G-A	c.191C>T	p.Pro64Leu	2.34E-04	VUS	VUS	(-0.01)	Significant Alteration of ESE/ESS motif ratio (-2)	No Impact	NES**
4-110687833-T-C	c.205A>G	p.Lys69Glu	2.39E-05	Not Reported	VUS	0.02	Potential Splicing Alterations: activation of cryptic acceptor site	Gain ESE & ESS	NES**
4-110687811-G-C	c.227C>G	p.Ala76Gly	Novel	VUS	VUS	0.01	No impact	No Impact	NES**
4-110685820-C-T	c.355G>A	p.Gly119Arg	4.25E-04	Conflicting Interpretations	LP	0.41	Significant Alteration of ESE/ESS motif ratio (-4)	Gain ESE Loss/Gain ESS	Exon 3 Skipping
4-109764547-C-T	c.472G>A	p.Gly158Arg	1.19E-05	Not Reported	VUS	(-0.01) & 0.30	Potential Splicing Alterations: activation of cryptic acceptor site	Gain ESE cryptic acceptor site	Exon 3 Skipping
4-110682801-T-A	c.530A>T	p.Asn177Ile	6.01E-05	VUS	VUS	(-0.03)	Significant Alteration of ESE/ESS motif ratio (-2)	No Impact	NES**
4-110682781-C-T	c.550G>A	p.Val184Met	Novel	VUS	VUS	0.01	No Impact	Gain ESS	NES**
4-110682761-C-A	c.570G>T	p.Glu190Asp	7.96E-06	VUS	VUS	(-0.02) & 0.01	Significant Alteration of ESE/ESS motif ratio (-7)	Loss ESE	NES**
4-110682703-C-T	c.628G>A	p.Ala210Thr	1.19E-05	Not Reported	VUS	(-0.10)	No Impact	Gain ESE	NES**
4-110681732-G-C	c.719C>G	p.Ala240Gly	2.55E-04	Conflicting Interpretations	VUS	(-0.07)	Significant Alteration of ESE/ESS motif ratio (-11)	Gain ESS	NES**
4-110681679-C-T	c.772G>A	p.Ala258Thr	1.17E-04	P/LP	LP	(-0.73) & 0.04	Alter WT Donor site; most probably affects splicing	Loss ESS	Exon 5 Skipping
4-110681506-G-A	c.803C>T	p.Ser268Leu	2.05E-06	VUS	VUS	(-0.11) & 0.01	Significant Alteration of ESE/ESS motif ratio (-4)	No Impact	NES**
4-110681503-C-A	c.806G>T	p.Gly269Val	Novel	Not Reported	VUS	(-0.02) & 0.01	Potential Splicing Alterations: activation of cryptic donor site	No Impact	NES**
4-110681450-C-T	c.859G>A	p.Gly287Arg	4.60E-05	Conflicting Interpretations	VUS	(-0.01) & 0.02	Potential Splicing Alterations: activation of cryptic acceptor site	Loss ESE	NES**
4-110670750-G-A	c.949C>T	p.Arg317Trp	2.12E-05	VUS	VUS	(-0.18)	No Impact	Loss ESE	NES**
4-110670749-C-T	c.950G>A	p.Arg317Gln	2.12E-05	VUS	VUS	(-0.36)	Potential Splicing Alterations: activation of cryptic acceptor site	Loss ESE	Exon 9 Skipping

4-110670693-G-C	c.1006C>G	p.Arg336Gly	2.00E-04	Not Reported	VUS	(-0.16) & 0.01	No Impact	Loss ESE	NES**
4-110670411-C-T	c.1111G>A	p.Gly371Ser	Novel	VUS	VUS	(-0.05) & 0.01	Potential Splicing Alterations: activation of cryptic acceptor site	Gain ESE	NES**
4-110670410-C-A	c.1112G>T	p.Gly371Val	1.90E-05	VUS	VUS	(-0.01) & 0.01	Potential Splicing Alterations: activation of cryptic donor site	Gain ESS	NES**
4-110667657-C-T	c.1150G>A	p.Ala384Thr	7.77E-05	VUS	VUS	0.01	No Impact	No Impact	NES**
4-110667618-C-A	c.1189G>T	p.Val397Leu	Novel	Not Reported	VUS	(-0.01)	No Impact	Loss ESE	NES**
4-110667617-A-T	c.1190T>A	p.Val397Glu	Novel	Not Reported	VUS	(-0.01) & 0.02	No Impact	Loss ESS	NES**
4-110667591-G-A	c.1216C>T	p.Arg406Cys	9.20E-05	VUS	VUS	(-0.01)	Significant Alteration of ESE/ESS motif ratio (-5)	Gain ESE	NES**
4-110667590-C-T	c.1217G>A	p.Arg406His	4.47E-03	B	B	(-0.01) & 0.02	Significant Alteration of ESE/ESS motif ratio (-3)	No Impact	NES**
4-110667561-T>G	c.1246A>C	p.Ile416Leu	6.98E-04	VUS	VUS	0.01	No Impact	No Impact	NES**
4-110667554-T-A	c.1253A>T	p.His418Leu	1.19E-05	Conflicting Interpretations	LP	0.01	Significant Alteration of ESE/ESS motif ratio (-7)	No Impact	NES**
4-110667524-T-C	c.1283A>G	p.Asn428Ser	3.98E-06	VUS	VUS	0.01	Potential Splicing Alterations: activation of cryptic acceptor/donor site	No Impact	NES**
4-110667465-G-A	c.1342C>T	p.Arg448Cys	7.16E-05	VUS	VUS	(-0.09) & 0.06	Significant Alteration of ESE/ESS motif ratio (-6)	No Impact	NES**
4-110667453-C-G	c.1354G>C	p.Ala452Pro	3.18E-05	Not Reported	VUS	(-0.11) & 0.10	Significant Alteration of ESE/ESS motif ratio (-6)	Loss ESE	NES**
4-110667378-C-G	c.1429G>C	p.Asp477His	1.99E-05	VUS	VUS	(-0.75) & 0.37	Alter WT Donor site; most probably affects splicing	Gain ESE; Loss ESS; Destroys donor site	Multiple ORF
4-110667377-C-G	c.1429+1G>C		2.83E-05	P/LP	P	(-0.77) & 0.41	Alter WT Donor site; most probably affects splicing	Loss ESS; Destroys donor site	Multiple ORF

*Collected from the gnomAD database (GRCh37/hg19; ENSG00000205403.8). Data from ClinVar and Franklin collected in 4/2024.

#Positive values indicate predict splice gains, while negative values indicate predicted splice losses. Scores are from 0 to ±1, with 0 meaning no impact on splicing and ±1 meaning impact on splicing.

$HSF = human splicing finder

**NES = No effect on splicing

Supplementary Table 4. **ELISA vs. RID FI Measurements**

Sample	ELISA*	RID**
1	28.07	25.28
2	24.50	24.73
3	35.43	28.43
4	36.56	26.89
5	35.05	29.02
6	29.26	25.81
7	47.70	41.39
8	36.14	30.61
9	41.00	35.86
10	22.82	19.74
11[±]	10.28	23.11
12[±]	12.42	26.57
13[±]	10.20	29.16
14[±]	13.61	29.44

[±]Highlights are the individuals carry a heterozygous SNP in *CFI*, chr4:110667590-C-T (c.1217G>A, p.Arg406His, hg19). Results by ELISA in these individuals show significantly reduced levels compared to those obtained by RID, suggesting that the variant c.1217G>A affects the binding of either the capturing or detecting antibody in ELISA.

* ELISA = Enzyme-linked immunoassay; reference range: 18-44 mg/L

** RID = Radial immunodiffusion; reference range: 16-40 mg/L
